# Predictors of suboptimal adherence to isoniazid preventive therapy among adolescents and children living with HIV

**DOI:** 10.1371/journal.pone.0243713

**Published:** 2020-12-17

**Authors:** Alexander W. Kay, Neil Thivalapill, Donald Skinner, Gloria Sisi Dube, Nomathemba Dlamini, Bulisile Mzileni, Patricia Fuentes, Pilar Ustero, Lisa V. Adams, Anna M. Mandalakas

**Affiliations:** 1 Department of Pediatrics, Baylor College of Medicine and Texas Children’s Hospital, Global Tuberculosis Program, Section of Global and Immigrant Health, Houston, Texas, United States of America; 2 Baylor College of Medicine Children’s Foundation, Mbabane, Eswatini; 3 Department of Biological Sciences, Columbia University, New York, New York, United States of America; 4 HIV AIDS STDs and TB, Human Sciences Research Council, Cape Town, South Africa; 5 National Tuberculosis Control Program, Ministry of Health, Mbabane, Eswatini; 6 Section of Infectious Disease and International Health, Geisel School of Medicine, Dartmouth College, Hanover, New Hampshire, United States of America; University of Southern California, UNITED STATES

## Abstract

This study identified factors associated with adherence to a 6-month isoniazid preventive therapy (IPT) course among adolescents and children living with HIV. Forty adolescents living with HIV and 48 primary caregivers of children living with HIV completed a Likert-based survey to measure respondent opinions regarding access to care, quality of care, preferred regimens, perceived stigma, and confidence in self-efficacy. Sociodemographic data were collected and adherence measured as the average of pill counts obtained while on IPT. The rates of suboptimal adherence (< 95% adherent) were 22.5% among adolescents and 37.5% among the children of primary caregivers. Univariate logistic regression was used to model the change in the odds of suboptimal adherence. Independent factors associated with suboptimal adherence among adolescents included age, education level, the cost of coming to clinic, stigma from community members, and two variables relating to self-efficacy. Among primary caregivers, child age, concerns about stigma, and location preference for meeting a community-health worker were associated with suboptimal adherence. To determine whether these combined factors contributed different information to the prediction of suboptimal adherence, a risk score containing these predictors was constructed for each group. The risk score had an AUC of 0.87 (95% CI: 0.76, 0.99) among adolescents and an AUC of 0.76 (95% CI: 0.62, 0.90), among primary caregivers suggesting that these variables may have complementary predictive utility. The heterogeneous scope and associations of these variables in different populations suggests that interventions aiming to increase optimal adherence will need to be tailored to specific populations and multifaceted in nature. Ideally interventions should address both long-established barriers to adherence such as cost of transportation to attend clinic and more nuanced psychosocial barriers such as perceived community stigma and confidence in self-efficacy.

## Introduction

The End TB Strategy, adopted by the World Health Organization (WHO) in 2015, called for a 90% reduction in TB related deaths and an 80% reduction in TB incidence by 2030 [1]. However, these goals are unlikely to be met without additional focus on TB prevention [2]. The WHO has placed an emphasis on preventive therapy with multiple guidelines and global reports over the last 5 years [3, 4]. Despite these efforts, the WHO 2020 Global TB Report estimates isoniazid preventive therapy (IPT) initiation among people living with HIV (PLHIV) globally at 50%, with no data on IPT completion [5]. In Eswatini (formerly Swaziland), a WHO high-burden HIV/TB country, 65% of people newly enrolled in HIV care during 2019 initiated IPT [5]. These data are not age disaggregated and data on IPT initiation and completion are unavailable for child and adolescent populations living with HIV.

IPT implementation is limited by a number of structural factors including inefficient clinical programming and poor health infrastructure [6, 7]. Extensive research demonstrates the clinical efficacy of IPT [8], the impact of different preventive regimens on adherence [9–11], and the benefits of creating a national policy on IPT [12, 13]. Less work has explored the patient and caregiver perspectives on IPT, a knowledge gap requiring elucidation to help bridge the divide between policy and practice [7].

There are limited data surrounding IPT uptake and completion in children and adolescents living with HIV as compared to adults [14]. Data that exists suggest that predictors of adherence to IPT in children and adolescents living with HIV are distinct from adults [15, 16].

Here we report the outcomes of a survey of primary caregivers of children and adolescents prescribed IPT and conducted at a large pediatric HIV and TB clinic in Eswatini. The survey and subsequent analysis interrogate the associations between a number of potential barriers to IPT completion and suboptimal adherence.

## Methods

The study took place between January 2016 and August 2017 at the HIV and TB Center of Excellence (COE) of the Baylor College of Medicine Children’s Foundation-Eswatini, which are free standing integrated facilities sharing a common campus. Written informed consent was obtained from all adult PCGs, including those of adolescent participants. Assent was obtained from children over 10 years of age. Ethics approval was obtained from Baylor College of Medicine Children’s Foundation Eswatini, the Eswatini Ethics Committee (MH/599C/FWA 000 15467/IRB 000 9688), the Baylor College of Medicine Institutional Review Board (H-34451), Houston, Texas, USA and the Dartmouth-Hitchcock Medical Center’s Committee for the Protection of Human Subjects (STUDY00028423), Hanover, NH, USA. The COE is located in Mbabane, Eswatini, and serves patients from both urban and rural areas. The COE provides care and treatment for over 2000 children, adolescents, and primary caregivers living with HIV. From this population, 50 adolescents aged 13–19 and 49 primary caregivers of children 0–12 years were referred to participate in the study if they had been prescribed IPT at a recent visit. Following completion of written informed consent, participants completed a written survey administered in SiSwati; a study officer read the questionnaire to illiterate participants.

The surveys for adolescents and primary caregivers contained 64 and 72 items, respectively. The survey questions were adapted, after discussions with key stakeholders, from a previously published survey validated in a similar population [17, 18]. Questions were designed to assess a range of factor categories contributing to adherence; including, knowledge of TB disease and prevention, attitudes towards the clinic, stigma from various sources, self-efficacy, and physical and financial barriers to adherence. Adherence to the regimen was determined by the average of pill counts performed during the IPT course and was dichotomized to suboptimal adherence (less than 95%) and optimal adherence (95% or greater). Ten adolescents and 1 child of a primary caregiver had missing pill counts and were excluded from the analysis, resulting in a final cohort of 40 adolescents and 48 primary caregivers.

Univariate logistic regression was used to model the change in the odds of suboptimal adherence by factors relating to one of the categories previously mentioned. Given the small sample sizes of our cohorts, *a priori*, a Type I error rate of 10% was used for the univariate logistic regression analysis. While this may increase the chances of a false positive association between a survey question and suboptimal adherence, a more conservative Type I error rate may have precluded identification of truly meaningful associations that were limited in evidence because of low power.

Risk scores for suboptimal adherence were then constructed for each group using the variables identified through logistic regression analyses and through subject matter knowledge to prevent the inclusion of correlated predictors such as age and education. Area under the Receiver-Operator Characteristic curve (AUC) was estimated non-parametrically for each risk score in its respective cohort. All statistical analyses were conducted using Stata 15 (College Station, TX USA).

## Results

Tables 1 and 2 highlight demographic and socioeconomic characteristics of adolescents and primary caregivers, respectively. Among the 40 adolescent responders included in the analysis, 19 (47.5%) were female, while the majority of the 48 primary caregiver responders, 39 (81.3%) were female. The average age of the adolescent responders was 15.5 and the average age of the primary caregivers was 37.2, while the average age of the child for which they were responsible was 8.6 years. The vast majority of both groups used a commercial kombi (minibus) as their primary mode of transportation to the clinic (92.5% among adolescents and 91.7% among primary caregivers). Adolescents and primary caregivers had similar household characteristics with respect to the type of structure and the number of people in the household.

**Table 1:** Characteristics of adolescent respondents.

Variable	Category	N = 40 (%)
Age		15.5 (1.6)
Gender	Male	21 (52.5%)
	Female	19 (47.5%)
Religion	Anglican/Catholic	2 (5.0%)
	Evangelical/Charismatic	4 (10.0%)
	Zionist	12 (30.0%)
	Other	22 (55.0%)
Mode of Transportation	Walk	3 (7.5%)
	Kombi	37 (92.5%)
Distance to Clinic in Minutes*		81.50 (60.6)
Highest Education Grade Level*		7.75 (1.9)
Household Construction	Brick	28 (70.0%)
	Stick & Mud	12 (30.0%)
Number of People in Household*		5.07 (2.9)
TB Contact	Yes	2 (5.0%)
	No	37 (92.5%)
	Don’t Know	1 (2.5%)
Taking IPT	No	37 (92.5%)
	Yes	3 (7.5%)
Months on IPT*		1.00 (0.0)
Previous Awareness of IPT	Yes	23 (57.5%, 95% CI: 40.9%, 73.0%)
Adherence*		97.3 (11.5)
Adherence Categories	95% or Greater	31 (77.5%)
	Less than 95%	9 (22.5%)

*Indicates continuous measure with SD reported.

**Table 2:** Characteristics of primary caregiver respondents.

Variable	Category	N = 48 (%)
Age*		37.2 (12.2)
Age of Child*		8.6 (2.7)
Gender	Male	9 (18.8%)
	Female	39 (81.3%)
Religion	Anglican/Catholic	2 (4.2%)
	Evangelical/Charismatic	3 (6.3%)
	Zionist	10 (20.8%)
	Other	33 (68.8%)
Mode of Transportation	Walk	0 (0.00%)
	Kombi	44 (91.7%)
	Personal Car	4 (8.3%)
Distance to Clinic in Minutes*		64.27 (37.1)
Highest Caregiver Education Grade Level*		9.02 (3.0)
Societal Participation	Attending School	4 (8.3%)
	Regular Work Context	16 (33.3%)
	Irregular Work Context	6 (12.5%)
	Neither attending school nor working	22 (45.8%)
Household Construction	Brick	37 (77.1%)
	Stick and Mud	11 (22.9%)
People in Household*		4.94 (2.2)
Child had a TB Contact	Yes	10 (20.8%)
	No	38 (79.2%)
Child Currently Taking IPT	No	42 (87.5%)
	Yes	6 (12.5%)
Months on IPT*		1.3 (0.5)
Previous Awareness of IPT	Yes	7 (14.6%, 95% CI: 6.1%, 27.8%)
Adherence*		85.0 (34.8)
Adherence Categories	95% or Greater	30 (62.5%)
	Less than 95%	18 (37.5%)

*Indicates continuous measure with SD reported.

Thirty-seven adolescents (92.5%) and 38 children of primary caregivers (79.2%) did not report a known TB contact and 3 (7.5%) adolescents and 6 (12.5%) children of primary caregivers reported already being initiated on IPT at the time the survey was conducted. Adolescents were much more aware of IPT prior to the time of the survey than primary caregivers (57.5%, 95% CI: 40.9%, 73.0% vs. 14.6%, 95% CI: 6.1%, 27.8%). The average adherence among adolescents was 97.3% (SD: 11.5) while the average adherence among children of primary caregivers was 85.0% (SD: 34.8). When adherence was categorized using a 95% or greater threshold for optimal adherence, 9 adolescents (22.5%) and 18 (37.5%) children of primary caregivers had suboptimal adherence.

Among adolescents, seven questions of the survey were associated with suboptimal adherence by univariate logistic regression analysis (Table 3). Older individuals were less likely to have suboptimal adherence (OR: 0.41 95% CI: 0.20, 0.88) and a similar odds reduction was observed for the association between educational attainment and suboptimal adherence (OR: 0.54, 95% CI: 0.30, 0.99). Concerns about stigma impacted adherence; adolescents who believed that their neighbors would think they had HIV if they took an IPT pill every day had an increased odds of suboptimal adherence (OR: 2.16, 95% CI: 0.97, 4.80). Regarding self-efficacy, the more confident adolescents were in their ability to both pick up medicines at the COE monthly and in their ability to be examined by a TB nurse monthly, the less likely they were to have suboptimal adherence (OR: 0.50, 95% CI: 0.27, 0.92, and OR: 0.56, 95% CI: 0.30, 1.04, respectively). Preference of medication regimen also appeared to be associated with suboptimal adherence—adolescents who preferred one type of medicine daily for six months (i.e. six months of daily isoniazid), consistent with the regimen that was offered, had lower odds of suboptimal adherence compared to those who preferred two types of medicine once a week for three months (i.e. three months of weekly isoniazid and rifapentine) (OR: 0.55, 95% CI: 0.28, 1.10). Finally, cost was also determined to be associated with the odds of suboptimal adherence—adolescents who did not find the cost of coming to the COE prohibitive had reduced odds of suboptimal adherence (0.52, 95% CI: 0.26, 1.03).

**Table 3:** Selected univariate logistic regression for variables included in model for adherence < 95% among adolescents.

Variables	OR	P-Value	95% Confidence Interval
Increasing Age	0.41	0.02	(0.20, 0.88)
Neighbors May Associate HIV with IPT	2.16	0.06	(0.97, 4.80)
Cost of clinic visits is not prohibitive	0.52	0.06	(0.26, 1.03)
Feels able to pick up medicine at clinic monthly	0.50	0.03	(0.27, 0.92)
Feels able to be examined by TB nurse monthly	0.56	0.07	(0.30, 1.04)
Prefers daily to weekly medication	0.55	0.09	(0.28, 1.10)

HIV; Human Immunodeficiency Virus, IPT; isoniazid preventive therapy, TB; tuberculosis.

Among primary caregivers, similar associations were identified between survey questions and suboptimal adherence (Table 4). For example, the older the age of the child under the care of the primary caregiver, the less likely they were to have suboptimal adherence (OR: 0.79, 95% CI: 0.63, 1.01). Potential stigma from neighbors were also identified among primary caregivers. Primary caregivers who believed that their neighbors would think that their child was sick or had TB if they gave them an IPT pill every day, the greater the odds of suboptimal adherence for the child (OR: 2.46, 95% CI: 1.10, 5.51 and OR: 1.75, 95% CI: 0.91, 3.37, respectively). Finally, primary caregivers who preferred not to meet in a social setting or in the community health worker’s home were much more likely to have children with suboptimal adherence (4.28, 95% CI: 1.21, 15.15).

**Table 4:** Selected univariate logistic regression for variables included in model for adherence < 95% among primary caregivers.

Variables	OR	P-Value	95% Confidence Interval
Increasing age of Child	0.79	0.06	(0.63, 1.01)
Neighbors may think the child is sick if taking IPT	2.46	0.03	(1.10, 5.51)
Neighbors my think the child has TB if taking IPT	1.75	0.10	(0.91, 3.37)
Prefers not to meet CHW in a social setting or at the CHW’s home	4.28	0.02	(1.21, 15.15)

HIV; Human Immunodeficiency Virus, IPT; isoniazid preventive therapy, TB; tuberculosis, CHW; community health worker.

Among adolescents, the score was constructed with equal weights of categorized age, the cost variable, the two self-efficacy variables regarding picking up medications and being examined monthly, and the stigma variable of neighbors suspecting that the respondent had HIV if they took IPT. Education was excluded to prevent the incorporation of similar information to age in the score. The AUC of this score was 0.87 (95% CI: 0.76, 0.99) with respect to prediction of suboptimal adherence. Among primary caregivers, the score was categorized with equal weights of categorized age of the child, the two stigma variables of neighbors suspecting that the respondent’s child was sick or had TB if the child was adherent to their IPT regimen, and whether the respondent was comfortable meeting the CHW in a social setting or at their home. The AUC of this score was 0.76 (95% CI: 0.62, 0.90).

## Discussion

Despite the WHO recommending IPT as part of a comprehensive package of HIV care and treatment a decade ago, routine clinical implementation of IPT for PLWH has been suboptimal [5]. Adequate adherence among children and adolescents is often circumscribed to the research context and WHO notification data illustrate poor IPT uptake throughout the African region, suggesting a disconnect between research and clinical practice [19, 20]. Hence, clinical approaches to IPT delivery should be informed by identification of social and behavioral drivers of adherence among both primary caregivers and adolescents [17, 18]. Here, we identify patient characteristics and beliefs associated with suboptimal adherence to IPT, which represent potential targets of adherence interventions and may help to identify caregivers and adolescents who will require more support to complete IPT.

The demographic and socioeconomic characteristics demonstrated in Tables 1 and 2 indicate that adolescents had more awareness of IPT prior to survey administration compared to primary caregivers, suggesting the need for counseling primary caregivers in particular on the benefits of IPT. Children of primary caregivers had lower rates of adherence than adolescents; however, more adolescents than children of primary caregivers were excluded due to missing outcomes. The adherence outcomes are unlikely to be missing completely at random, and selection bias may preclude meaningful statistical comparisons between these two groups.

In both groups, overlapping and unique predictors of suboptimal adherence were identified via univariate logistic regression analysis. Specifically, stigma from neighbors, HIV stigma for adolescents and TB/general illness stigma for primary caregivers, were predictive of suboptimal adherence in both groups. Primary caregivers had lower adherence if they preferred not to meet in a social setting or in the CHW’s home. We postulate that fear of community-derived stigma against HIV and TB underlies these preferences; hence, adherence interventions for IPT must also address patient perceptions of stigma toward these underlying conditions. These observations are consistent with adult data from South Africa, which suggests that stigma is associated with both IPT and ART [21]. Additionally, the younger the adolescent or the child of the primary caregiver, the more likely the odds of suboptimal adherence. This suggests the need for age-appropriate adherence interventions focused on young adolescents and primary caregivers of young children. This relationship is consistent with ART adherence data for infants living with HIV, but conflicts with the relationship between age and nonadherence to ART identified in other adolescent cohorts [22]. Further investigation is required to better contextualize this relationship with adherence to IPT, and how this may be different to ART adherence, in the adolescent population.

Among adolescents alone, self-efficacy, as measured by confidence in one’s ability to pick up medicine monthly and be examined by the TB nurse monthly, was an important predictor of suboptimal adherence. The relationship between self-efficacy and adherence to ART is well-documented in the literature [23]. Our data validate the impact of self-efficacy on adherence to IPT as well, suggesting that some of the underpinnings of suboptimal adherence may be similar for both ART and IPT in PLHIV. These observations support the integration of IPT counseling and ART counseling, with an emphasis on promoting the confidence of adolescents to achieve healthy behaviors that include not only adherence, but attending their clinic appointments as well. Finally, the cost of coming to the COE was determined to be a significant predictor of suboptimal adherence to IPT. Transport costs have been demonstrated to impact adherence to HIV and TB treatment [24–26], and indicates the need to avoid additional clinical visits associated with IPT. More broadly, these observations demonstrate the need to increase access clinics for adolescents who are often managing competing responsibilities between their education, household responsibilities, and work.

Although six months of daily IPT was the only regimen being offered at the COE, adolescents in this study who preferred six months of a daily medication had higher rates of adherence than those who preferred two medications given weekly. This observation highlights the importance of differentiated service delivery and providing options that match patient lifestyles and competing priorities [27]. This observation does not indicate that six months of IPT will be superior to once weekly 3HP, if both options are available.

The risk scores presented here demonstrate the collective utility that each set of predictors has in identifying those who are most likely to have suboptimal adherence to IPT regimens. This compilation of predictors represents a starting place for future study and a framework of factors that clinicians should consider when initiating IPT in a child or adolescent. That they differ in their construction indicates that the drivers of adherence are necessarily different between adolescents and primary caregivers and that subsequent interventions designed to increase adherence must be population-specific. The heterogeneity in performance of the risk scores also suggests that adherence, an act largely driven by behavioral and psychosocial factors, is more difficult to predict among the children of primary caregivers than adolescents. This may be explained by the difficulty in gauging the drivers of adherence in a child through a proxy such as, in this case, a caregiver.

A threshold of 95% was used to determine suboptimal adherence given the recent literature on the importance of high adherence to TB regimens in achieving favorable outcomes [28, 29], and similar thresholds typically used for ART adherence. Adherence targets for IPT have generally been less stringent, but many of the definitions for successful IPT completion have been derived through expert opinion rather than evidence. The threshold of 95% used in this study revealed epidemiologically plausible and consistent associations between a number of survey variables and suboptimal adherence, suggesting that this cutoff may in fact be appropriate for our data and context. Further, while this may represent overfitting of the data, the consistency between the results obtained and previous evidence on adherence barriers in similar settings suggests that the outcome of suboptimal adherence is still captured in spite of a more conservative threshold.

There are a number of limitations to this study. In particular, a small cohort size likely minimized the power of the study to detect true associations and conversely, a higher Type I error threshold may have increased the false-positive rate between predictor and the outcome of suboptimal adherence. In addition to concerns of internal validity, our patients were recruited from an underlying population that is generally healthy, is well supported by clinicians at the COE, and has high rates of health-seeking behaviors; hence our results may not be applicable to all adolescents living with HIV and primary caregivers of children living with HIV in the SSA region.

## Conclusion

Our analysis demonstrates the important roles that financial barriers, community-derived stigma, and self-efficacy play in predicting suboptimal adherence to IPT regimens among adolescents and primary caregivers. Our data indicate that these two populations are substantively different with respect to the drivers of their adherence; therefore, interventions designed to increase adherence should be age-appropriate, population-specific, and barrier-targeted. Our findings highlight the need for enhanced community engagement to reduce stigma and increase community support for patients living with HIV. This analysis of barriers from the perspective of the adolescents and primary caregivers should inform strategies to improve IPT implementation in Eswatini and other resource-poor settings.

## Supporting information

S1 File (PDF)
